# Predicting Long-Term Deformation of Soundproofing Resilient Materials Subjected to Compressive Loading: Machine Learning Approach

**DOI:** 10.3390/ma13184133

**Published:** 2020-09-17

**Authors:** Seungbum Koo, Jongkwon Choi, Changhyuk Kim

**Affiliations:** 1 The MathWorks Inc., Natick, MA 01760, USA; skoo@mathworks.com; 2 Department of Civil and Environmental Engineering, Hongik University, Seoul 04066, Korea; jongkwon.choi@hongik.ac.kr; 3 Department of Civil, Architectural, and Environmental Engineering, The University of Texas at Austin, Austin, TX 78712, USA; 4 Korea Institute of Civil Engineering and Building Technology, Ilsan 10223, Korea

**Keywords:** resilient material, long-term deformation, floor impact sound, machine learning

## Abstract

Soundproofing materials are widely used within structural components of multi-dwelling residential buildings to alleviate neighborhood noise problems. One of the critical mechanical properties for the soundproofing materials to ensure its appropriate structural and soundproofing performance is the long-term compressive deformation under the service loading conditions. The test method in the current test specifications only evaluates resilient materials for a limited period (90-day). It then extrapolates the test results using a polynomial function to predict the long-term compressive deformation. However, the extrapolation is universally applied to materials without considering the level of loads; thus, the calculated deformation may not accurately represent the actual compressive deformation of the materials. In this regard, long-term compressive deformation tests were performed on the selected soundproofing resilient materials (i.e., polystyrene, polyethylene, and ethylene-vinyl acetate). Four levels of loads were chosen to apply compressive loads up to 350 to 500 days continuously, and the deformations of the test specimens were periodically monitored. Then, three machine learning algorithms were used to predict long-term compressive deformations. The predictions based on machine learning and ISO 20392 method are compared with experimental test results, and the accuracy of machine learning algorithms and ISO 20392 method are discussed.

## 1. Introduction

Multi-dwelling residential buildings are currently being constructed in areas with high population densities since these buildings can offer many houses within a limited urban space. This type of building allows residents access to workplaces and conveniences as well as economic benefits to society. Though multi-dwelling residential buildings have various advantages, they also have inherent problems due to their structural characteristics: shared floor, ceiling, and walls. In multi-dwelling residential buildings, any source of noise generated within a unit can be transmitted to adjacent units through those shared components. Specifically, the floor impact sound is generally more severe than others and can lead to troubles or even lawsuits between neighbors. 

Many countries have established standards for quantifying floor impact sounds. The U.S. Department of Housing and Urban Development (HUD) [1] has categorized five usages, and three steps for indoor rooms and floor impact sounds, respectively. European countries have similar standards to the United States regarding lightweight floor impact sounds. For example, Denmark, Norway, Finland, Estonia, and Poland stipulate the minimum standard of lightweight impact sound at 53 dB, while Sweden, Slovakia, and Latvia stipulate the standard at 58 dB [2]. Japan has individual standards for lightweight, and heavyweight impact sounds [3], and the performance of the dwelling is classified into five levels according to the sound insulation performance based on the evaluation criteria curve. The Republic of Korea has a standard to prevent noise using floor impact sound isolation structures [4]; the structure has to limit lightweight and heavyweight impact sounds under 58 and 50 dB. The standard proposes three types of floor structures based on these decibel limits. The thickness of the concrete slab, resilient material, finishing, and mortar was considered to control the level of impact.

To mitigate the floor impact sounds, the use of resilient materials within floor slabs has recently become popular in research and construction practice [5,6]. These materials are typically defined to have characteristics such as impact sound-absorbing and vibration damping. Theoretically, increasing the floor slab thickness can alleviate the transmission of floor impact sound and vibration. However, this remedy will also unnecessarily increase the self-weights of the floor slabs, and result in increased structural demands for the entire structural system; eventually, the construction cost will inevitably go up. Therefore, resilient materials exhibiting superior soundproofing performance without sacrificing structural performance have been widely implemented in modern building construction. Various resilient materials such as expanded polystyrene (EPS), ethylene-vinyl acetate (EVA), and polyethylene (P.E.) have been developed and applied to numerous building construction. To maximize the effectiveness of such resilient materials, many standards, including ISO, have been developed to characterize the material properties of resilient materials such as density, dynamic stiffness, loss coefficient, residual strain, and heat conductivity [7]. Many of these current standards are only based on short-term loading experimental results [6,8,9]. For example, ISO 29770 [10] and KS F 2873 [11] limit the residual strains based on 11-min experimental results under different loading conditions. However, floor resilient materials are continuously exposed to loads for its service life. The material properties per the standards using a short-term loading history may not be adequate to predict the long-term performance of floor resilient materials [12]. Also, the nonhomogeneity of the materials and the slab deflection caused by the service loading conditions could lead to cracking of the entire floor system, leading to a reduction in the sound insulation performance [13]. Such a shortage makes the research on the long-term deflection of sound resilient structural material necessary.

Experiment-based studies are intrinsically data-driven. Classically, researchers gather the experimental data and extract an empirical formula or a model that can best describe the behavior of the material under question within the collected information. However, as the behavior of the material becomes highly nonlinear, the formula becomes more complex and harder to use. To ease the usage and eventually be free of a visible formula, we can utilize the emerging machine learning approach to estimate the material behavior. The data-driven property of material behavior study also makes it an excellent candidate to apply the machine learning strategy.

Applications of machine learning algorithms have shown reasonably good results in various engineering fields. Artificial neural networks (ANN) and linear regression have been used to predict the performance of a diesel engine [14]. The use of ANN provided more accurate test results than the use of linear regression modeling. Machine learning algorithm approaches were reviewed to estimate concrete material properties such as flow, slump, strength, and serviceability [15]. Backpropagation neural network and self-organization features were used to estimate concrete properties. This study reported that the repeated random subsampling method was recommended to reduce statistical bias. A multilayer feed-forward ANN creep behavior was developed to enhance asphalt mixtures creep compliance predictions [16]. Hot mix asphalt (HMA) creep compliance behavior can be predicted using an ANN model in a short amount of time with a low error rate. A first-order multiple linear regression was developed to predict the permanent deformation behavior of the plant produced asphalt concrete mixtures [17]. The produced linear regression model was compared to an existing model, and the results showed an improved performance of the developed model in the study. ANN and adaptive neuro-fuzzy inference system models were proposed to predict the shear strength of grouted reinforced concrete masonry walls [18]. The ANN and neuro-fuzzy inference system models well predicted the test results and obtained improved performance as compared to the existing empirical models. ANN could well describe the behavior of the magnetorheological elastomer base isolator [19]. Also, many ANN models have been considered and proposed to predict the compressive strength of concrete [20,21].

It must be noted here that the machine learning approach used for this purpose still falls into the regression analysis. Regression analysis is a statistical process to estimate the relationship between dependent variables and independent variables. The machine learning approach is doing the regression analysis that the conventional way—non-computer-based—does, except for the computational efficiency.

This paper presents a technique to predict the long-term compressive deformation of resilient materials using novel machine learning algorithms: K-nearest neighbors (KNN), regression tree (RT), and artificial neural networks (ANN). A compressive creep test results over 350 and 500 days were used to train and evaluate the machine learning algorithms. First, the above-mentioned machine learning algorithms were trained using the actual data set. Then, the predictions obtained from the trained algorithms were compared to the prediction by ISO 20392 procedure. ISO 20392 provides a means to evaluate the compressive creep of resilient material [22]. This standard, however, allows us to measure the compressive deformation for the first 90 days. The long-term compressive responses of resilient materials after the first 90 days are estimated based on Findley’s equations [23]. The accuracy and reliability of Findley’s equations are yet questionable in this situation because of two reasons. First, Findley’s equations are developed based on the test results of plastic laminates. Those equations do not account for the variable shape of resilient materials. Second, the predictions are typically based on relatively short-term loading tests (90-day compressive creep tests).

The two estimations are compared with the measured data points, and the long-term deformation prediction ability will be evaluated.

## 2. Experimental Study

### 2.1. Specimen Details

**Figure 1:**
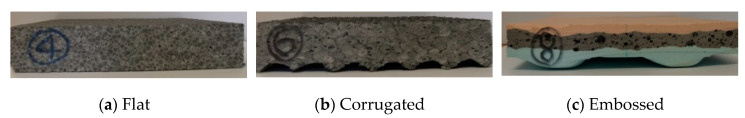
Bottom shape of the resilient materials.

Six types of resilient materials widely used in construction practice were selected to investigate their long-term compressive deformation performance. The test was performed following ISO 20392. The specimens were expanded polystyrene 1 (EPS1), expanded polystyrene 2 (EPS2), polyethylene (P.E.), and ethylene-vinyl acetate (EVA). The densities of the specimens ranged from 12 to 25 kg/m^3^ for EPS1, EPS2, and P.E., and it was 59.3 kg/m^3^ for EVA-59-E. As shown in Figure 1, the patterns of the resilient materials’ bottom surfaces were flat, corrugated, or embossed. The base dimensions of all specimens were 150 mm × 150 mm based on the ISO 20392 requirement, and the thickness of the specimens was chosen to be 30 mm, which is typically used in construction practice. Table 1 shows a summary of the specimen properties, such as the representative material properties, thickness, and configuration of the bottom surface.

A universal testing machine (UTM) was used to measure the elastic modulus of the resilient materials. A steel plate was placed on the resilient materials to prevent eccentricity. The deformation-controlled load was monotonically applied to the specimens at a speed of 0.02 mm/s. The specimens were loaded until the deformation reached 50% of the initial thickness. The load-deformation relationship of the specimens showed near-linear responses, whereas EVA-59-E showed bilinear responses. The reason for the bilinear responses was due to the shape of the EVA-59-E. In the case of EVA-59-E, unlike other specimens, EVA-59-E consisted of two types of cross-sections: flat and embossed. To evaluate the elastic modulus of each resilient material, the tangent moduli measured at a specific strain of 0.30 was defined as the elastic moduli of the respective material. It should be noted that the elastic moduli of EVA-59-E were evaluated at specific strains of 0.17 and 0.30, because of an obvious bilinear response of the load-deformation relationship.

### 2.2. Loading Protocol and Instrumentations

To measure the long-term compressive deformation of the resilient materials under sustained loads, the load levels of 40, 80, 250, and 500 N were applied to 24 specimens over 350- and 500-day periods. These four loading conditions were selected to represent various loading conditions of residential buildings. The load levels of 40 and 80 N, representing the permanent loads (i.e., dead loads), were applied to the specimens over 350-day periods. The load levels of 250 N and 500 N, which induce 0.011 and 0.022 MPa of stresses on the resilient materials, respectively, are representatives of heavy appliances or furniture such as refrigerators and pianos in the household. In the cases of 250 N and 500 N, the loads were applied to the specimens over 500-day periods.

**Figure 2:**
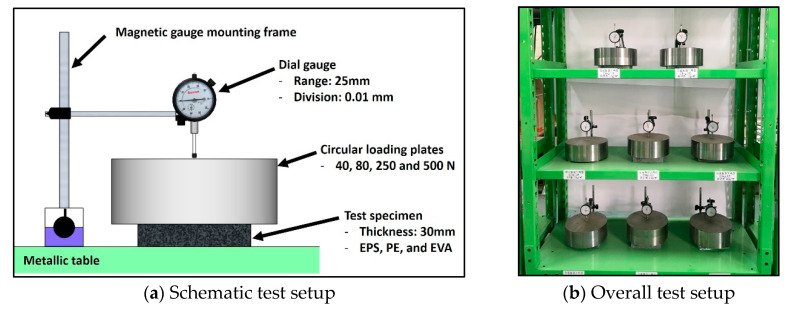
Test setup.

As shown in Figure 2, the loads were applied using a set of circular loading plates. Enlarged circular loading plates with a diameter of 220 mm were fabricated to apply sustained loads uniformly distributed over the entire area of the test specimen. To measure the axial deformation of the specimen, a dial gauge was installed at the centroid of the top-loading plate using a gauge frame magnetically-mounted on the reference test table on which the entire test setup was placed. The dial gauge used to measure the deformation of the resilient materials has 0.01 mm accuracy. To accurately measure the initial deformation due to the loading plate installation, the dial gauge was installed immediately after placing the load plate. Since the thickness of the specimens after 1 min of loading is defined as an actual deformation according to ISO 20392, the dial gauge was installed within 1 min of loading.

The long-term deformations of the resilient materials were measured for more than 500 days. The first measurement was taken at 1 min after loading, then consecutive measurements were made at 10 min, 30 min, 1 h, 5 h, 10 h, 1 day, 2 days, and 3 days after the specimens were loaded. Between 3 and 30 days, the deformation was measured every 3 days. After 30 days, the deformation was measured every ten days. The measuring intervals are summarized in Table 2. 

## 3. Learning Algorithms

In this section, the learning algorithms used in this article are briefly introduced. Three algorithms are considered, namely, distance-weighted k-nearest-neighbor regression (KNN), regression tree (RT), and artificial neural network (ANN).

### 3.1. Distance Weighted KNN Regression

The k-nearest neighbor (KNN) algorithm is intrinsically suitable for such increasing relationships because it involves neighboring data points and, therefore, sensitive to the local slope. For a given set of data $S=\left\{\left(x^{i},y^{i}\right)|1\leq i\leq N\right\}$, where vector $x^{i}$ denotes a collection of *i*-th input features and scalar $y^{i}$ denotes the corresponding output label, distance weighted KNN regression predicts the label $y^{q}$ for a query $x^{q}$ as the distance weighted average of *k* nearest neighbors of $x^{q}$, where *k* is a preselected parameter. In other words,

(1) $$y^{q}=\frac{\sum_{j=1}^{k}w_{j}y^{j}}{\sum_{j=1}^{k}w_{j}}$$

where,

(2) $$w_{j}=\frac{1}{||x^{q}-x^{j}||}$$

KNN is a type of instance-based learning algorithm. The learning in this algorithm is done by simply storing the provided training set *S* in the computer’s memory space. Then, in the later querying step, the algorithm loads the *k* number of nearest neighbors of the query point $x^{q}$ and returns the predicted label $y^{q}$ by the predefined mechanism. This method does not construct any model that spans the entire instance space but instead finds a local approximation in the neighborhood of the query point, which, in turn, has a significant advantage when the model is expected to be very complicated. However, the query time may be very long, since the algorithm must investigate every data point in *S* to find the nearest *k* neighbors in the worst case, and may have large memory costs since the prediction must inquire of *S*. Additional regression details such as equations for KNN can be found in Kim et al. [19]. 

### 3.2. Regression Tree

The regression tree (RT) algorithm if the ranges are adequately divided since the local slopes will be better expressed. RT builds a binary decision tree with the given training set *S*. Starting from the root node, the algorithm tracks down the branches of the upside-down tree with the given query point $x^{q}$ until reaching the terminal node, also known as the leaf node. Depending on the split value that determines which branch to track down, there are subcategories of trees. The most commonly used norm is the correlation coefficient between each feature in $x^{i}$ and the corresponding label $y^{i}$; however, one can also randomly select any feature in $x^{i}$ to decide which branch to follow. The leaf size is a user tunable parameter that must be carefully selected. Smaller leaf size leads to overfitting and degrades the prediction accuracy. Bigger leaf size, on the other hand, tends to average all the data in *S* and ultimately gives no meaningful information. It is recommended to either sweep through every leaf size to seek an optimal leaf size or implement some strategy such as bagging or boosting to introduce regularity. Learning in RT is implemented as building the tree with given *S*, and *S* can be forgotten after the learning is finished since the querying is done with the RT, not with *S*. Such implementation expedites the query time compare to KNN; however, the whole tree must be rebuilt if one wants to add additional data points.

In this article, RT with the highest correlation coefficient is implemented, and the algorithm is as follows:

**Step 1** Let $x^{i}$, i=1,⋯,N be the length *d* vectors where $x_{j}^{i}$, j=1,⋯,d indicates the *j*-th feature of *i*-th instance among *N* instances in *S*. The corresponding label is a scalar value $y^{i}$. Then, for every feature *j* in $x^{i}$, i=1,⋯,N, calculate the correlation coefficient with the labels $y^{i}$, i=1,⋯,N, and select the feature *j* that has the highest correlation coefficient.
**Step 2** Let *j* be the highest correlated feature to $y^{i}$. Take the median among $x_{j}$ as the value to binary split the node and form two children nodes. Denote the split value as *c*.
**Step 3** Training set *S* has now been split into two subsets with respect to *c*, namely $S_{1}=\left\{\left(x^{i},y^{i}\right)|x_{j}^{i}<c\right\}$ and $S_{2}=\left\{\left(x^{i},y^{i}\right)|x_{j}^{i}\geq c\right\}$ where $S=S_{1}\cup^{\;}S_{2}$ and $S_{1}\cap^{\;}S_{2}$ is an empty set. Repeat Steps 1 and 2 for subsets $S_{1}$ and $S_{2}$ until the number of elements in all of the subsets becomes less than or equal to the predefined leaf size.

Once the tree is built, for a given query point $x^{q}$, the algorithm tracks down the tree from the root node and returns the prediction $y^{q}$ when it reaches the leaf node. $y^{q}$ can be taken as the mean of $y^{i}$’s in the destination leaf node, since there can be multiple $y^{i}$’s in a leaf. The value other than the mean of $y^{i}$’s can also be considered, whatever is appropriate. Additional regression details such as equations for RT can be found in Kim et al. [19].

### 3.3. Artificial Neural Network

The artificial neural network (ANN) algorithm is robust when the data points have random change over the entire range, such as abrupt jumps. The ANN algorithm possesses a mechanism to include all patterns appearing in the whole range. Thus, the ANN is presumed to be less suitable for the present application than the other two methods. ANN superficially mimics the biological nerve system, although many details are neglected in implementation. When there is an input $x^{i}$ and output $y^{i}$, many computational units called neurons are arranged in layers between inputs and outputs. Each neuron in a layer is linked to other neurons in other layers, and ultimately to the inputs and outputs with some weights. These layers and weights are usually out of consideration after the training is completed since the one we find useful is the trained model, not the optimum layer configuration and weights; therefore, we say that these layers are hidden. The number of neurons in the hidden layers and the number of layers in a model is typically determined through trial and error, which is a significant shortcoming of this algorithm. However, once an optimum configuration is found, the querying is implemented relatively swiftly.

**Figure 3:**
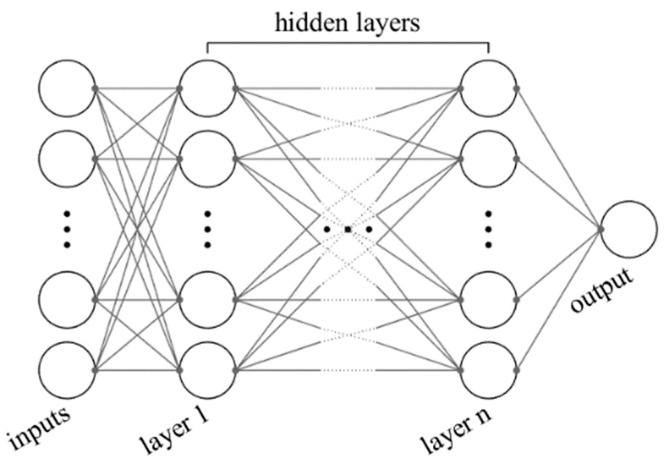
General schematic of an artificial neural network (ANN).

The training in this algorithm is the process of finding an optimal set of weights that connect the neurons in layers. Depending on the method of optimizing the weights to fit the given dataset *S* at best, the direction of the data flow, and other detailed implementation specifics, ANN can be categorized into several different types. In this article, we relied on by far the most popular method, called the feed-forward, backpropagation method, where the data moves in one-way from the input to the output through hidden layers, and the error terms at the end of each trial, also known as an epoch, propagate back to the lower layer, and the weights are iteratively adjusted in every epoch until they converge. A schematic of this is shown in Figure 3.

**Figure 4:**
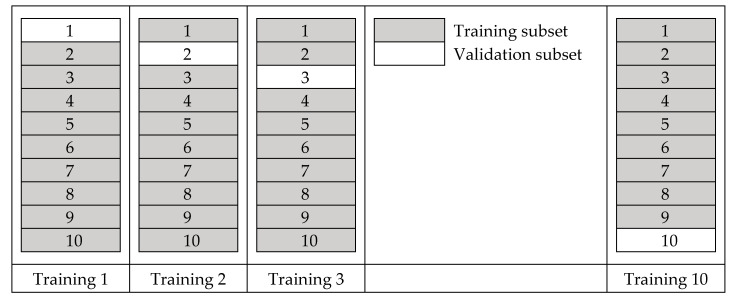
Combinations of the training set and validation set.

For this particular application, the model landed at two hidden layers, where each layer consists of 50 neurons. Sigmoid activation function was used that gave the highest fidelity compared to the others, which were also chosen by trial and error. The dataset was divided into ten subsets, where nine were used for training, and one was used for validation. There are ten possible combinations to group the training set and validation set in this approach, and all ten combinations were used for training the ANN model. (Figure 4.) Additional regression details such as equations for ANN can be found in Kim et al. [19].

## 4. Results and Analysis

In this section, the predictions of long-term compressive deformation responses using three machine learning algorithms are presented. Those algorithms were trained using the experimental test results presented in Section 2. The accuracy of the predictions made by the trained models is evaluated by comparing them with the experimental observations and predictions based on ISO 20392. Specifically, the authors focused on the capabilities of the trained algorithms regarding the long-term deformation predictions compared to the estimations per ISO 20392. Lastly, the feasibility of each machine learning algorithm for application in the long-term compressive deformation prediction was discussed.

### 4.1. Test Results

**Figure 5:**
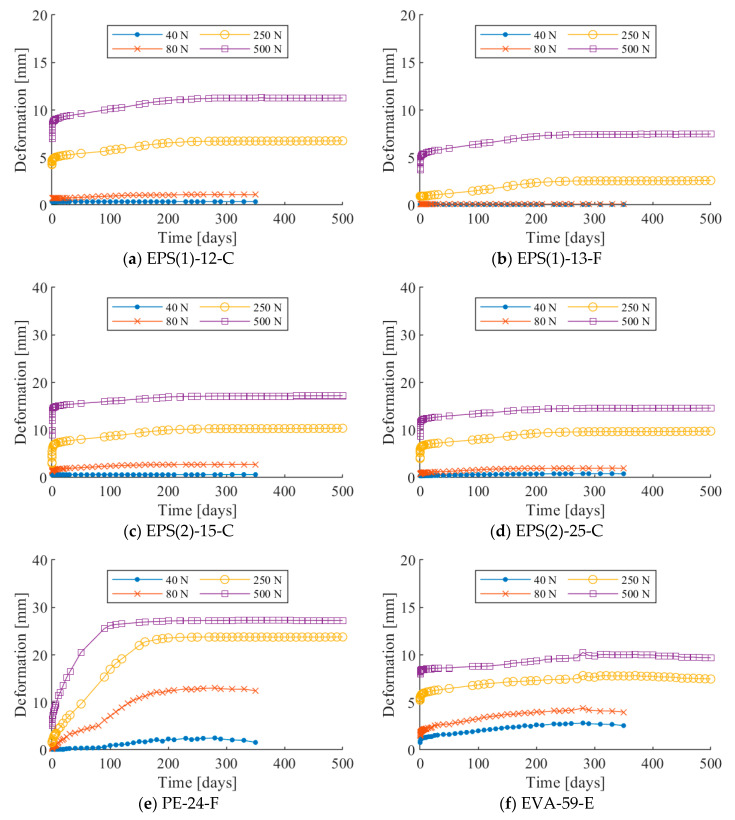
Long-term compressive deformation.

The long-term compressive deformation tests on six soundproofing resilient materials were conducted. Their deformations were monitored for periods of 350 days (for the specimens loaded at both 40 and 80 N) and 500 days (for the specimens loaded at 250 and 500 N). Figure 5 shows the deformations of each specimen under various loading conditions over time. It can be seen that the long-term deformations of the models asymptotically approach their horizontal asymptotes.

The long-term deformation varied depending on the mechanical properties, specimen dimensions, and loading situations of resilient materials. The larger the applied load, the greater the increment in long-term deformation was observed; however, they were not proportional to the applied loads.

PE-24-F deflected the most in all loading cases, while EPS 2 deflected more than EPS 1. In particular, the PE-24-F specimen showed deformations of 23.9 and 27.2 mm under 250 and 500 N loads, respectively, which is more than 80% deformation as compared to the initial thickness (30 mm). The deformation in the specimens with the corrugated bottom shape was larger than that of flat bottom shape. In the resilient materials with identical materials and bottom shape, the deformation was larger for the specimen with lower density. Across all specimens, deformations were observed when the applied load was 80, 250, and 500 N; however, no deformation could be recognized as long-term deformation when they were lightly loaded (40 N).

The deformation trend in all loading cases was similar across all specimens. The increment in deformations was large at the initial stage of loading and gradually decreased until they sufficiently reached their asymptotes. This trend was more apparent when the specimens were subject to heavier loads: 250 and 500 N.

### 4.2. ISO 20392 and Trained Models’ Predictions

Here, the long-term deformations of resilient materials estimated by ISO 20392 and by our trained learning algorithms are compared to the actual measured data, and their estimation quality and accuracy are discussed.

ISO 20392 calculates the long-term compressive deformation using Equation (1). The input parameters required for the calculation are brought from the experiment settings. The equation uses information on a period up to the first 90 days only.

(3) $$X_{t}=X_{0}+mt^{b}$$

where, $m=10^{a},\;a=y_{m}-bx_{m},\;b=\frac{\sum^{\;}x_{t}y_{t}-\frac{\sum^{\;}x_{t}\sum^{\;}y_{t}}{n}}{\sum^{\;}x_{t}^{2}-\frac{{\left(\sum^{\;}x_{t}\right)}^{2}}{n}}$

In this equation, *X_t_* indicates the deformation at time *t*, *X*_0_ is the initial deformation, *x_m_* and *y_m_* are averages of *x_t_* and *y_t_*, respectively, *x_t_* is time (log*t*), and *y_t_* = creep (log*X_ct_*, *X_ct_* = *X_t_* − *X*_0_).

Various *k* values for KNN, tree depth, and leaf size for RT, and hidden layer configuration and choice of activation functions were considered to identify the best-trained models with the minimum root mean squared error (RMSE). KNN utilized three different distance weighting: No distance weighting, weighting by 1/distance, and weight by 1-distance. The minimum total weight of the instances in a leaf of RT was two. In the case of KNN, the value of the weighted KNN was four, with one over distance weighting. The tree depth of RT was limited to 10, and two hidden layers where each layer consists of 50 neurons were used for ANN and iterated for 1000 epochs.

The correlation coefficients and various errors between the test data and trained models plus ISO 20392 are tabulated in Table 3 for comparison. The average of the correlation coefficients of KNN, RT, and ANN was 0.9973 in the in-sample test, which implies that the trained models reflect the test results very well. However, when the data are extrapolated beyond 350 or 500 days, the prediction by ANN deviates very far from the rational region, and so must be rejected.

KNN returned the most accurate result among the trained models, including ISO 20392; the correlation coefficient increased by 6.1%, and the RMSE decreased by 43.4% with respect to ISO 20392. The evaluation of ISO 20392 was also done with a full range of test data, while the equation takes only the first 90 days of observation for the calculation. Thus, the rest of the data by ISO 20392 up to 350 or 500 days is extrapolated, and the assessment is not strictly an in-sample test. Taking note of this fact, the accuracy assessment of ISO 20392 in terms of correlation coefficient and other error measurements gives a good sense of the long-term deformation prediction ability of ISO 20392. It turns out that ISO 20392 is the worst compared to the trained models, which indicates that ISO 20392 does not accurately predict the long-term behavior of the resilient materials, and the use of a learning algorithm is justified.

**Figure 6:**
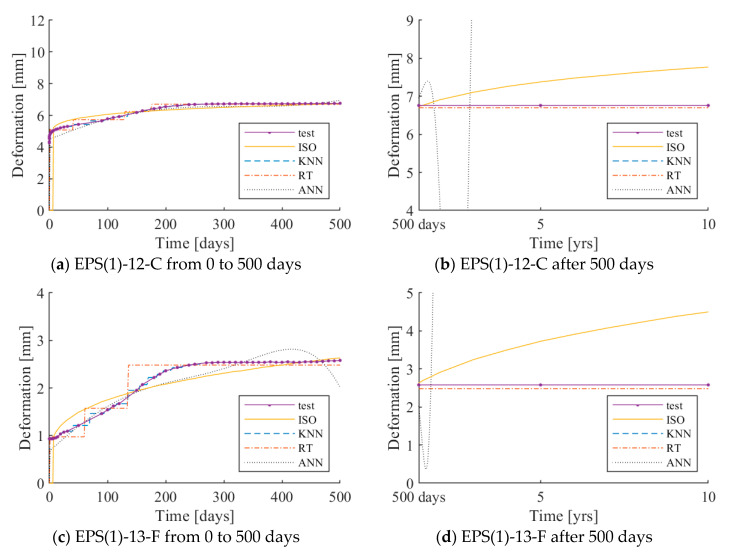

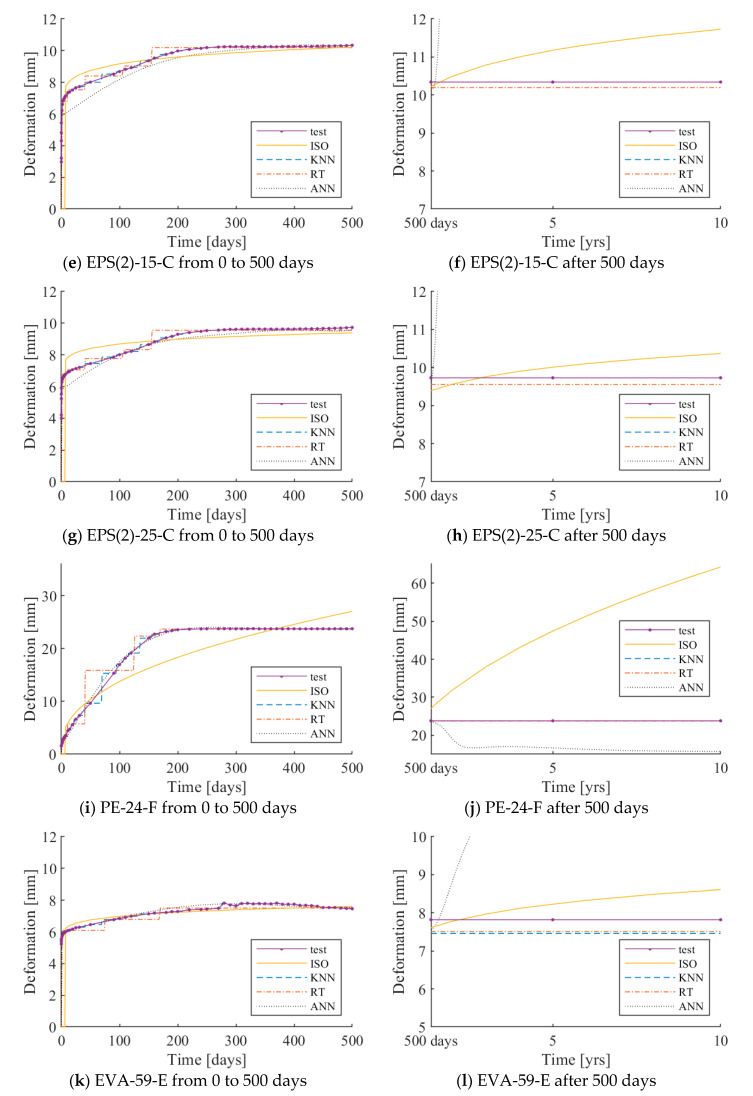
Long-term deformations as predicted by ISO 20392 (solid), k-nearest neighbor (KNN, dashed), regression tree (RT, dash-dot), and artificial neural networks (ANN, dotted) compared to the experimental data under 250 N loading.

**Figure 7:**
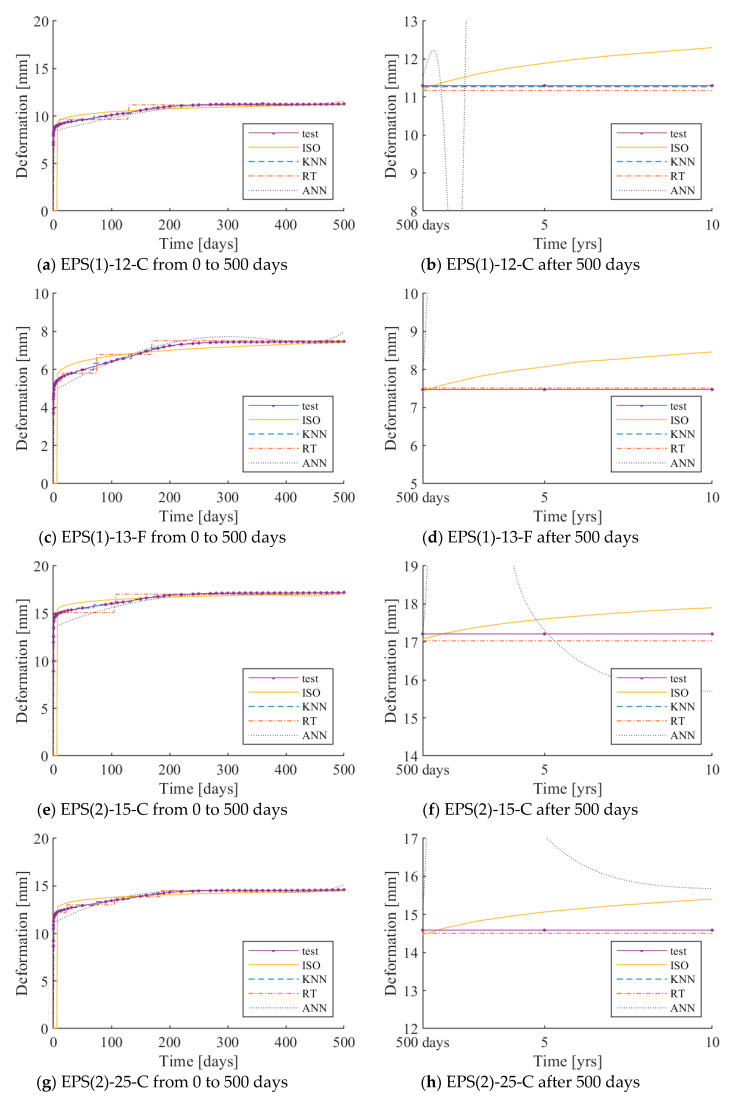

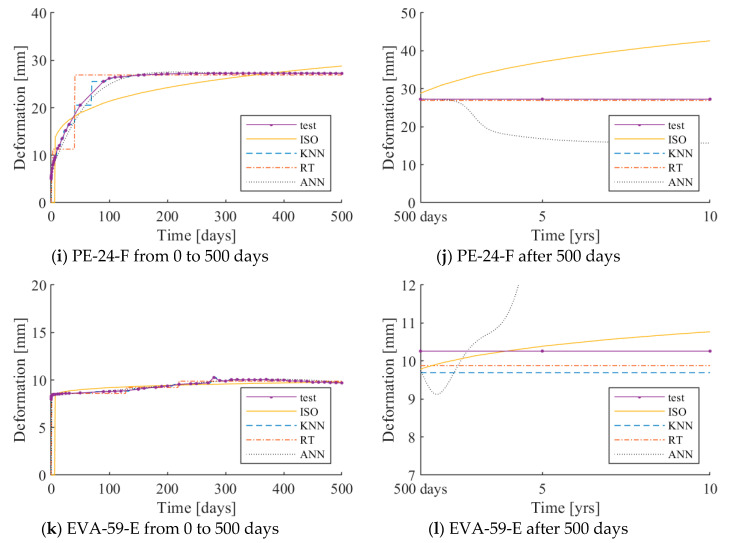
Long-term deformations as predicted by ISO 20392 (solid), KNN (dashed), RT (dash-dot), and ANN (dotted) compared to the experimental data under 500 N loading.

The test results under 250 and 500 N loadings are plotted with the ISO 20392 equation, and the predictions by each of the three learning algorithms are presented in Figure 6 and Figure 7, respectively. The same graphs for 40 and 80 N loadings can be found in the Appendix A. The x- and y-axes indicate the load application time and deformation of the resilient material, respectively. The solid curve with markers and dotted curve indicates the test results of the resilient materials and the ISO 20392 equation, respectively. The three different types of curves represent the deformation – time responses as predicted by the three learning algorithms. 

In the case of the prediction curve using ISO 20392, the predicted curve before 90 days was nearly the same as the test results, except for PE-24-F. Since the ISO 20392 equation depends on the test results of 90 days, large deformations occurring after 90 days are not reflected. The step-wise shape of the predictions observed in KNN and RT is due to the sparsely distributed test data, as the gradient of the deformation in time is very steep in the early stage of loadings. Increasing the parameter *k* in KNN can alleviate this trend; however, doing so increases RMSE. Therefore, the value of N.N. is set to one in this study. In the case of the ANN algorithm (multilayer perceptron), the algorithm predicts the test results well within the range of the actual test data. However, the prediction deviated sharply after 500 days.

Table 4 and Table 5 show the measured and predicted long-term deformations of the resilient materials under 250 and 500 N loadings, respectively. Since the KNN algorithm showed the smallest RMSE, the results of the deformation prediction of the KNN algorithm are shown in the table. 

The long-term deformation after 500 days, as calculated using ISO 20392, tends to increase gradually. In PE-24-F, the deformation estimated to occur after ten years was more than the initial thickness. Therefore, it is difficult to predict the long-term deformation of all of the resilient materials by the ISO equation. The long-term deformation using the KNN model did not increase after a certain point and converged. The tendency of the long-term deformation was nearly the same as the actual test results. This observation implies that the deformation prediction of the resilient materials using the KNN model is valid.

## 5. Conclusions

Whenever a new building material is considered in the design, it is important to understand their mechanical properties, structural performances, and long-term behaviors to ensure the safety of the structure during its service life. This article measured the long-term deformation of acoustically resilient material over 500 days. It then adopted a machine learning technique to build models that can accurately predict the resilient material’s asymptotic behavior over more extended periods.

Four loading conditions were considered, and six resilient material specimens were prepared to test the long-term deformations of the new materials. The measured data were then fed into three different machine learning algorithms, KNN, RT, and ANN, to train the models. Then, the predictions from each of the trained models were compared to the ISO 20392 prediction and the experimental test data to see how closely the trained models could estimate the long-term behaviors of resilient materials, and in turn, their potential for supplementing the shortcoming inherent in ISO 20392.

The main scope of this experimental study was the comparative analysis of the regression algorithms that could be applied to resilient materials among construction materials. Among the three different algorithms, the KNN algorithm was determined to predict the long-term behavior of the resilient materials usefully.

The conclusions of this study are summarized as follows:

(1) The compressive deformation of resilient materials varied depending on the material type, base shape, and density. Under the same long-term loading conditions and bottom patterns, both EPS 1 and EPS 2 deformed more than the others. The specimens with a corrugated or embossed bottom shape experienced more long-term compressive deformation. Also, the results of resilient materials with a higher density show that the amount of compressive deformation was less in denser resilient material.
(2) The predicted deformation responses using all three algorithms – KNN, RT, and ANN –could reasonably predict the actual experimental data, regardless of material properties. However, the predicted long-term responses using ANN showed significant deviations. Meanwhile, KNN and RT showed reasonable predictions for long-term deformation, which could supplement ISO 20392.
(3) The ISO 20392 model underestimates the long-term deformation response of PE-24-F. Such underestimation is because Findley’s equations (i.e., ISO 20392 model) were developed based on compressive creep tests on plastic laminates. As such, the ISO 20392 model is inappropriate for resilient materials which have variable shapes and nonhomogeneous material properties. However, machine learning techniques trained using an appropriate set of experimental test data are capable of accurately predicting long-term compressive deformation.

Extending the result of this literature can lead us to approach other novel constructional material’s behavior estimation using machine learning. We can also assess the results of structural analyses in two different paths: one with the structural member’s behavior calculated by the classical approach and the other with machine learning algorithms. Such comparison can provide us the confidence on the answers given by the machine learning approach, and ultimately provide the grounds for it to be used in practice.

## Figures and Tables

**Table 1:** Details of specimens.

Specimen	Material	Density (kg/m^3^)	Bottom Surface	Thickness (mm)	Specified Strain *	Elastic Modulus (MPa)
EPS(1)-12-C	Expanded Polystyrene 1	12.1	Corrugated	29.8	0.30	0.15
EPS(1)-13-F	Expanded Polystyrene 1	13.2	Flat	28.9	0.3	
EPS(2)-15-C	Expanded Polystyrene 2	15.4	Corrugated	30.9	0.30	0.11
EPS(2)-25-C	Expanded Polystyrene 2	25.5	Corrugated	31.9	0.30	0.12
PE-24-F	Polyethylene	24.0	Flat	31.0	0.30	0.16
EVA-59-E	Ethylene Vinyl Acetate	59.3	Embossed	30.2	0.17	0.11 **
					0.30	0.47 **

* Strain at which the elastic moduli were evaluated; ** Stress-strain response of ethylene-vinyl acetate (EVA) shows a bilinear relationship.

**Table 2:** Deformation measurement time.

Hour				Day																						
0.5	1	3	5	10	1	2	3	4	5	6	7	8	10	11	14	18	20	25	30	40	50	60	70	80	90	100

**Table 3:** Accuracy of predictions for each model.

Algorithm	Correlation Coefficient	Root Mean Square Error	Relative Absolute Error (%)	Root Relative Squared Error (%)
ISO 20392	0.9421	0.4546	32.9916	36.0530
KNN	0.9993	0.2572	1.5576	3.8750
RT	0.9949	0.6726	4.9822	10.1353
ANN	0.9977	0.4502	5.1224	6.7842

**Table 4:** Deformations of resilient materials under 250 N loading.

	Deformations of Resilient Materials under 250 N Loading (mm)																	
	EPS(1)-12-C			EPS(1)-13-F			EPS(2)-15-C			EPS(2)-25-C			PE-24-F			EVA-59-E		
Time (Day)	Test Result	ISO 20392	KNN	Test Result	ISO 20392	KNN	Test Result	ISO 20392	KNN	Test Result	ISO 20392	KNN	Test Result	ISO 20392	KNN	Test Result	ISO 20392	KNN
11	5.11	5.35	5.11	0.95	1.07	0.95	7.33	7.93	7.33	6.90	7.84	6.9	4.44	5.90	4.44	6.08	6.33	6.08
19	5.21	5.51	5.21	1.03	1.20	1.03	7.51	8.22	7.51	7.06	8.04	7.06	5.59	7.20	5.59	6.18	6.47	6.18
31	5.30	5.66	5.3	1.09	1.33	1.09	7.73	8.48	7.73	7.21	8.22	7.21	7.34	8.66	7.34	6.30	6.61	6.3
50	5.43	5.82	5.43	1.21	1.49	1.21	8.01	8.75	8.01	7.45	8.41	7.45	9.66	10.43	9.66	6.46	6.76	6.46
90	5.65	6.02	5.65	1.46	1.71	1.46	8.52	9.10	8.52	7.86	8.64	7.86	15.33	13.20	15.33	6.77	6.95	6.77
100	5.78	6.06	5.78	1.54	1.75	1.54	8.68	9.17	8.68	8.01	8.69	8.01	16.97	13.78	16.97	6.84	6.98	6.84
200	6.55	6.33	6.55	2.36	2.08	2.36	9.98	9.60	9.98	9.30	8.98	9.3	23.57	18.35	23.57	7.29	7.24	7.29
300	6.72	6.50	6.72	2.54	2.31	2.54	10.25	9.87	10.25	9.61	9.16	9.61	23.73	21.76	23.73	7.65	7.40	7.65
400	6.73	6.63	6.73	2.54	2.48	2.54	10.26	10.06	10.26	9.63	9.29	9.63	23.75	24.59	23.75	7.75	7.51	7.75
500	6.76	6.73	6.76	2.58	2.63	2.58	10.34	10.22	10.34	9.73	9.39	9.73	23.78	27.05	23.77	7.46	7.61	7.46
730	N/A	6.91	6.76	N/A	2.91	2.58	N/A	10.49	10.34	N/A	9.56	9.73	N/A	31.83	23.77	N/A	7.78	7.46
1460		7.26	6.76		3.50	2.58		11.00	10.34		9.90	9.73		43.00	23.77		8.12	7.46
2190		7.48	6.76		3.91	2.58		11.32	10.34		10.10	9.73		51.34	23.77		8.33	7.46
2920		7.64	6.76		4.23	2.58		11.55	10.34		10.25	9.73		58.25	23.77		8.49	7.46
3650		7.77	6.76		4.50	2.58		11.73	10.34		10.37	9.73		64.26	23.77		8.61	7.46

**Table 5:** Deformations of resilient materials under 500 N loading.

	Deformations of Resilient Materials under 500 N Loading (mm)																		
	EPS(1)-12-C			EPS(1)-13-F			EPS(2)-15-C				EPS(2)-25-C			PE-24-F			EVA-59-E		
Time (Day)	Test Result	ISO 20392	KNN	Test Result	ISO 20392	KNN	Test Result	ISO 20392	KNN	Test Result		ISO 20392	KNN	Test Result	ISO 20392	KNN	Test Result	ISO 20392	KNN
11	9.14	9.56	9.14	5.52	5.91	5.52	15.06	15.65	15.06	12.39		12.94	12.39	11.43	14.74	11.43	8.52	8.60	8.52
19	9.28	9.77	9.28	5.66	6.09	5.66	15.22	15.84	15.22	12.52		13.14	12.52	13.51	16.09	13.51	8.55	8.72	8.55
31	9.42	9.96	9.42	5.80	6.26	5.8	15.37	16.01	15.37	12.73		13.32	12.73	16.49	17.44	16.49	8.60	8.85	8.6
50	9.60	10.15	9.6	5.98	6.44	5.98	15.57	16.19	15.57	12.95		13.51	12.95	20.55	18.92	20.55	8.64	8.98	8.64
90	9.99	10.40	9.99	6.33	6.67	6.33	15.93	16.40	15.93	13.33		13.74	13.33	25.54	20.97	25.54	8.77	9.16	8.77
100	10.10	10.44	10.1	6.42	6.71	6.42	16.04	16.44	16.04	13.44		13.79	13.44	26.19	21.37	26.19	8.79	9.19	8.79
200	11.01	10.76	11.01	7.25	7.01	7.25	16.93	16.71	16.93	14.32		14.07	14.32	27.14	24.24	27.14	9.36	9.43	9.36
300	11.24	10.95	11.24	7.44	7.18	7.44	17.14	16.87	17.14	14.52		14.25	14.52	27.24	26.14	27.24	9.88	9.58	9.88
400	11.24	11.09	11.24	7.45	7.32	7.45	17.16	16.98	17.16	14.52		14.37	14.52	27.26	27.60	27.26	9.99	9.69	9.99
500	11.27	11.20	11.27	7.47	7.42	7.47	17.21	17.07	17.21	14.59		14.47	14.59	27.23	28.80	27.23	9.69	9.78	9.69
730	N/A	11.39	11.27	N/A	7.60	7.47	N/A	17.22	17.21	N/A		14.64	14.59	N/A	30.97	27.23	N/A	9.95	9.69
1460		11.77	11.27		7.96	7.47		17.51	17.21			14.96	14.59		35.47	27.23		10.27	9.69
2190		12.00	11.27		8.20	7.47		17.68	17.21			15.15	14.59		38.46	27.23		10.48	9.69
2920		12.16	11.27		8.33	7.47		17.81	17.21			15.29	14.59		40.75	27.23		10.64	9.69
3650		12.30	11.27		8.46	7.47		17.90	17.21			15.40	14.59		42.63	27.23		10.77	9.69

**Figure A1:**
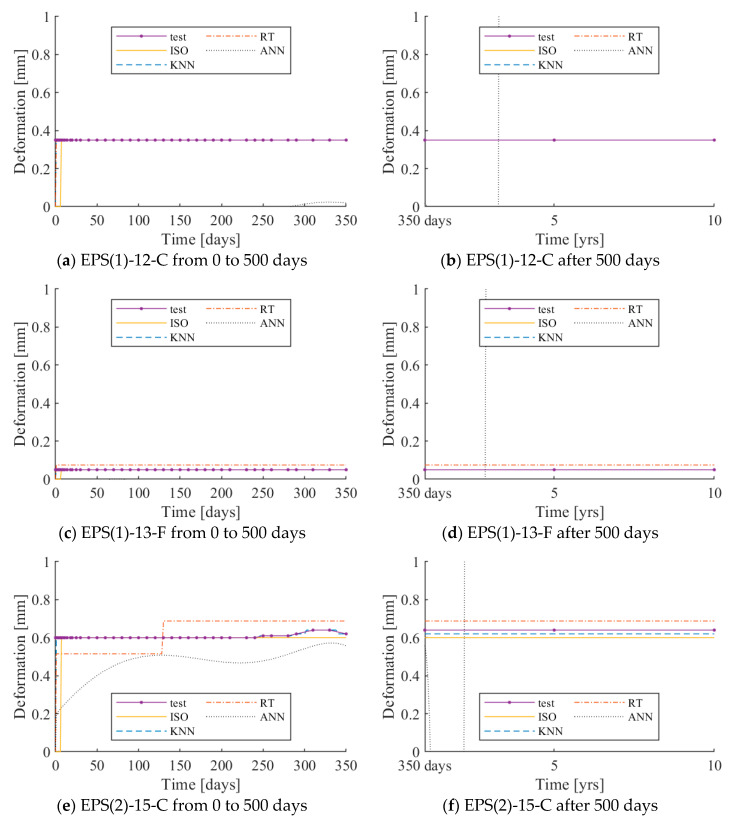

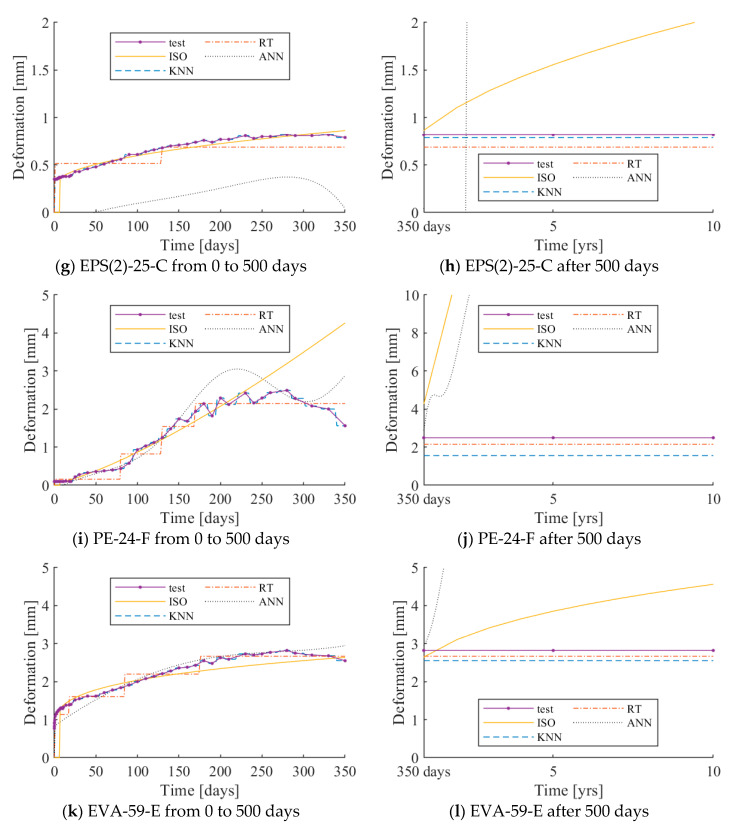
Long-term deformations as predicted by ISO 20392 (solid), KNN (dashed), RT (dash-dot), and ANN (dotted) compared to the experimental data under 40 N loading.

**Figure A2:**
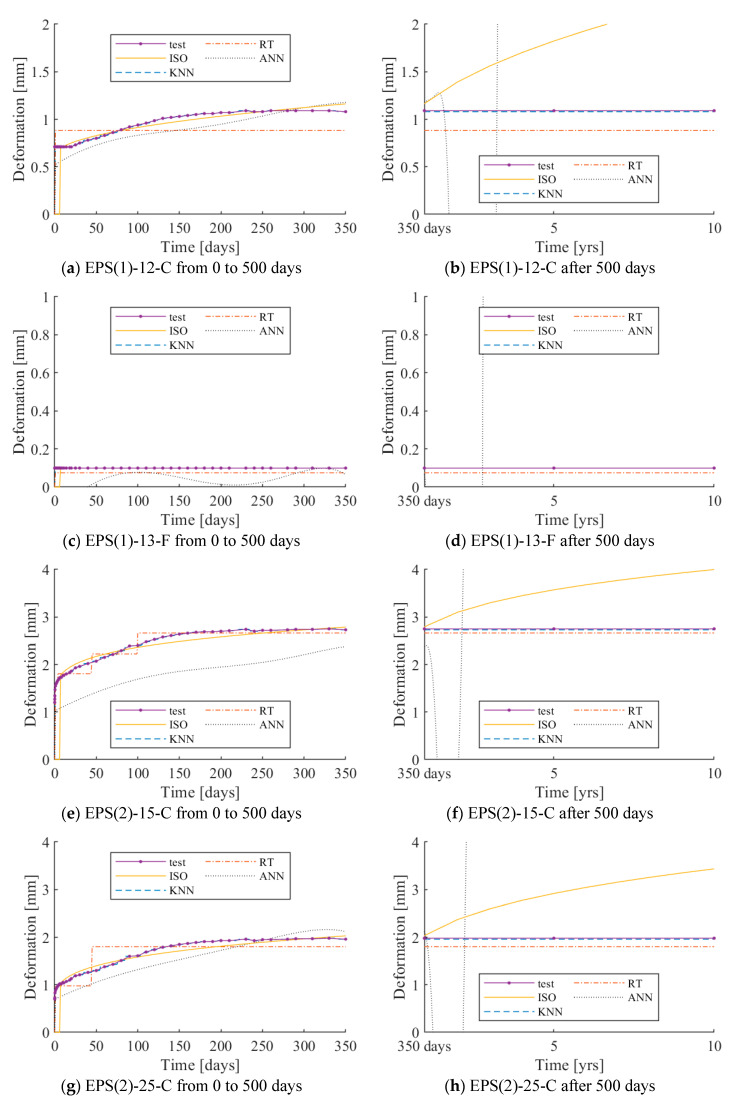

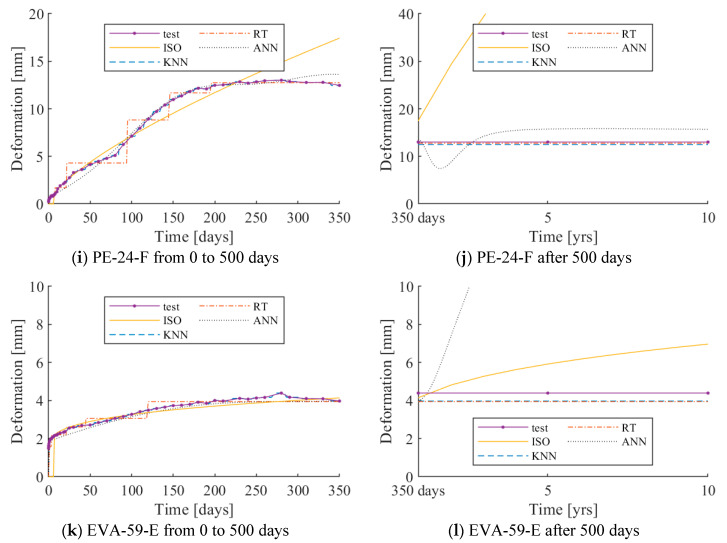
Long-term deformations as predicted by ISO 20392 (solid), KNN (dashed), RT (dash-dot), and ANN (dotted) compared to the experimental data under 80 N loading.

## Appendix A

**Table A1:** Deformations of resilient materials under 40 N loading.

	Deformations of Resilient Materials under 40 N Loading (mm)																	
	EPS(1)-12-C			EPS(1)-13-F			EPS(2)-15-C			EPS(2)-25-C			PE-24-F			EVA-59-E		
Time (Day)	Test Result	ISO 20392	KNN	Test Result	ISO 20392	KNN	Test Result	ISO 20392	KNN	Test Result	ISO 20392	KNN	Test Result	ISO 20392	KNN	Test Result	ISO 20392	KNN
10	0.35	0.35	0.35	0.05	0.05	0.05	0.60	0.60	0.6	0.38	0.38	0.38	0.10	0.09	0.1	1.29	1.36	1.29
20	0.35	0.35	0.35	0.05	0.05	0.05	0.60	0.60	0.6	0.39	0.42	0.39	0.10	0.15	0.1	1.40	1.52	1.4
30	0.35	0.35	0.35	0.05	0.05	0.05	0.60	0.60	0.6	0.43	0.46	0.43	0.28	0.22	0.28	1.55	1.63	1.55
40	0.35	0.35	0.35	0.05	0.05	0.05	0.60	0.60	0.6	0.46	0.48	0.46	0.33	0.30	0.33	1.62	1.71	1.62
60	0.35	0.35	0.35	0.05	0.05	0.05	0.60	0.60	0.6	0.51	0.53	0.51	0.38	0.47	0.38	1.71	1.85	1.71
80	0.35	0.35	0.35	0.05	0.05	0.05	0.60	0.60	0.6	0.56	0.57	0.56	0.44	0.66	0.44	1.84	1.95	1.84
100	0.35	0.35	0.35	0.05	0.05	0.05	0.60	0.60	0.6	0.61	0.60	0.61	0.93	0.87	0.93	2.00	2.04	2
200	0.35	0.35	0.35	0.05	0.05	0.05	0.60	0.60	0.6	0.77	0.73	0.77	2.29	2.08	2.29	2.63	2.34	2.63
290	0.35	0.35	0.35	0.05	0.05	0.05	0.62	0.60	0.62	0.81	0.81	0.81	2.28	3.34	2.28	2.75	2.53	2.75
350	0.35	0.35	0.35	0.05	0.05	0.05	0.62	0.60	0.62	0.79	0.86	0.79	1.56	4.27	1.56	2.55	2.64	2.55
730	N/A	0.35	0.35	N/A	0.05	0.05	N/A	0.60	0.62	N/A	1.11	0.79	N/A	11.09	1.56	N/A	3.11	2.55
1460		0.35	0.35		0.05	0.05		0.60	0.62		1.43	0.79		27.42	1.56		3.65	2.55
2190		0.35	0.35		0.05	0.05		0.60	0.62		1.67	0.79		46.59	1.56		4.03	2.55
2920		0.35	0.35		0.05	0.05		0.60	0.62		1.87	0.79		67.89	1.56		4.32	2.55
3650		0.35	0.35		0.05	0.05		0.60	0.62		2.05	0.79		90.92	1.56		4.56	2.55

**Table A2:** Deformations of resilient materials under 80 N loading.

	Deformations of Resilient Materials under 80 N Loading (mm)																	
	EPS(1)-12-C			EPS(1)-13-F			EPS(2)-15-C			EPS(2)-25-C			PE-24-F			EVA-59-E		
Time (Day)	Test Result	ISO 20392	KNN	Test Result	ISO 20392	KNN	Test Result	ISO 20392	KNN	Test Result	ISO 20392	KNN	Test Result	ISO 20392	KNN	Test Result	ISO 20392	KNN
10	0.71	0.71	0.71	0.10	0.10	0.1	1.77	1.83	1.77	1.05	1.09	1.05	1.27	1.45	1.27	2.18	2.29	2.18
20	0.71	0.75	0.71	0.10	0.10	0.1	1.86	1.96	1.86	1.13	1.20	1.13	2.28	2.32	2.28	2.37	2.52	2.37
30	0.75	0.78	0.75	0.10	0.10	0.1	1.96	2.05	1.96	1.21	1.28	1.21	3.30	3.07	3.3	2.59	2.67	2.59
40	0.78	0.81	0.78	0.10	0.10	0.1	2.02	2.12	2.02	1.26	1.35	1.26	3.61	3.75	3.61	2.69	2.79	2.69
60	0.83	0.85	0.83	0.10	0.10	0.1	2.15	2.22	2.15	1.38	1.45	1.38	4.47	4.99	4.47	2.84	2.98	2.84
80	0.89	0.88	0.89	0.10	0.10	0.1	2.29	2.30	2.29	1.51	1.52	1.51	5.09	6.11	5.09	3.06	3.13	3.06
100	0.94	0.91	0.94	0.10	0.10	0.1	2.40	2.36	2.4	1.61	1.59	1.61	7.12	7.15	7.12	3.27	3.26	3.27
200	1.07	1.03	1.07	0.10	0.10	0.1	2.70	2.58	2.7	1.93	1.81	1.93	12.47	11.70	12.47	4.00	3.70	4
290	1.09	1.11	1.09	0.10	0.10	0.1	2.74	2.72	2.74	1.97	1.95	1.97	12.87	15.25	12.87	4.17	3.98	4.17
350	1.08	1.16	1.08	0.10	0.10	0.1	2.73	2.79	2.73	1.96	2.03	1.96	12.46	17.44	12.46	3.97	4.14	3.97
730	N/A	1.39	1.08	N/A	0.10	0.1	N/A	3.10	2.73	N/A	2.37	1.96	N/A	29.49	12.46	N/A	4.82	3.97
1460		1.70	1.08		0.10	0.1		3.45	2.73		2.77	1.96		48.44	12.46		5.62	3.97
2190		1.93	1.08		0.10	0.1		3.68	2.73		3.04	1.96		64.78	12.46		6.17	3.97
2920		2.13	1.08		0.10	0.1		3.85	2.73		3.26	1.96		79.62	12.46		6.60	3.97
3650		2.30	1.08		0.10	0.1		4.00	2.73		3.44	1.96		93.44	12.46		6.97	3.97
